# A Case of Primary Subglottic Malignant Melanoma with a Successful Surgical Treatment

**DOI:** 10.1155/2014/968926

**Published:** 2014-06-08

**Authors:** Shahzad Ahmad, Mahmoud Abdelghany, Curtis Goldblatt, Owen Stark, Nicholas Masciotra

**Affiliations:** ^1^Department of Medicine, Conemaugh Memorial Medical Center, Johnstown, PA 15905, USA; ^2^Department of Anatomic and Clinical Pathology, Conemaugh Memorial Medical Center, Johnstown, PA 15905, USA; ^3^Department of Radiology, Conemaugh Memorial Medical Center, Johnstown, PA 15905, USA; ^4^Department of Otolaryngology, Conemaugh Memorial Medical Center, Johnstown, PA 15905, USA

## Abstract

Primary subglottic malignant melanoma is a very rare and underdiagnosed neoplasm. We are reporting a case of primary malignant melanoma of subglottic mucosa in a 78-year-old woman who presented to our hospital with shortness of breath and hoarseness of voice. Laryngoscopy and excisional biopsy along with immunoreactivity to S-100 and human melanoma black-45 (HMB-45) confirmed the diagnosis. The patient was treated with laryngectomy followed by radiotherapy. Five years following surgical treatment, she continues to be asymptomatic. To our knowledge, there is only one reported case of primary malignant melanoma of subglottic mucosa in the medical literatures.

## 1. Introduction

Although most melanomas are cutaneous in origin, primary malignant melanoma does occasionally arise from noncutaneous tissues that contain melanocytes, such as leptomeninges, uvea, and gastrointestinal, respiratory, and genitourinary tracts [1]. The least common of all the aforementioned sites is the subglottic mucosa of larynx. There are less than 60 cases of primary malignant melanoma of the larynx [2] and only one case of primary subglottic melanoma reported in the medical literatures [1].

## 2. Case

**Figure 1:**
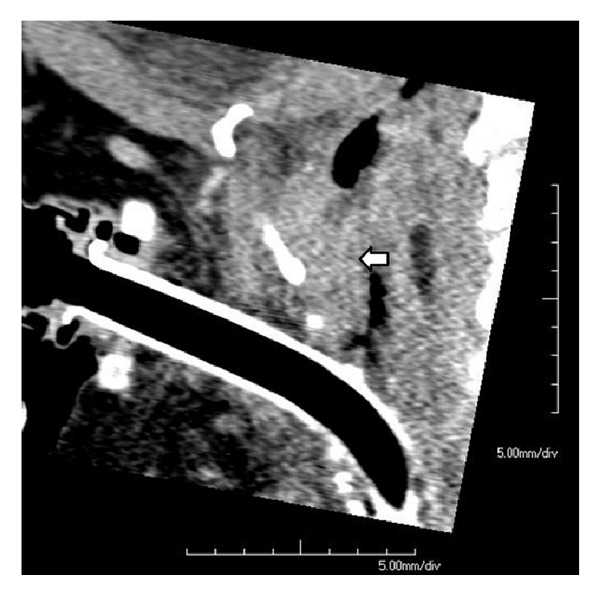
CT scan (sagittal image) of the neck with contrast performed after the initial tracheostomy demonstrates an ovoid mass (arrow) obstructing nearly the entire lumen of the airway. The mass measures 22 mm craniocaudally by 11 mm anteroposteriorly.

**Figure 2:**
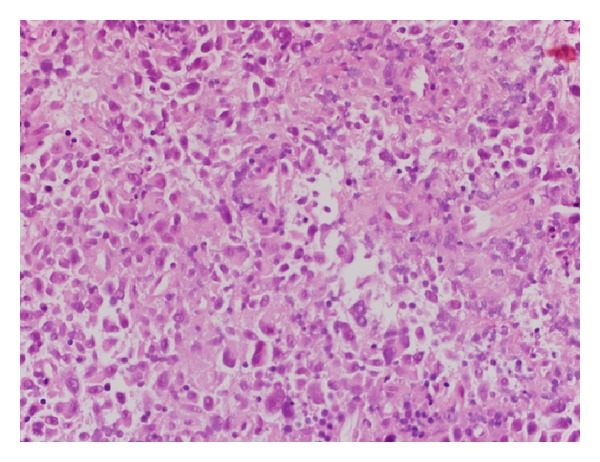
Hematoxylin and eosin (H&E) stained 40x objective photomicrograph shows discohesive malignant neoplasm with enlarged hyperchromatic pleomorphic nuclei and eosinophilic nucleoli and surrounding pale cytoplasm.

**Figure 3:**
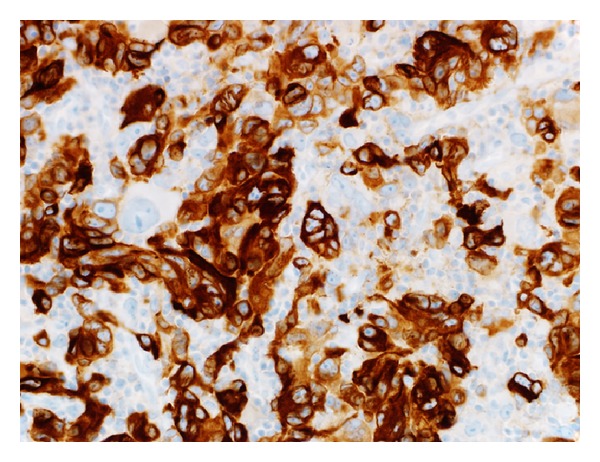
Melanin A stained 40x objective photomicrograph shows malignant cells stains strongly positive for melanin A.

**Figure 4:**
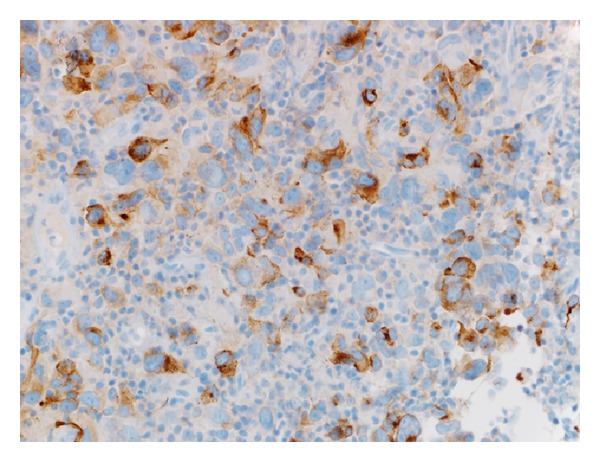
Human melanoma black-45 (HMB-45) stained 40x objective photomicrograph shows malignant cells stains strongly positive for HMB-45.

A 78-year-old white woman, with no significant past medical history, presented to our hospital because of progressively worsening dyspnea and hoarseness of voice for two months. She denied any other symptoms including dysphagia, odynophagia, and otalgia. At first, her symptoms were attributed to chronic obstructive pulmonary disease (COPD), and she was started on oxygen therapy and multiple medications for COPD, but her symptoms kept worsening. On presentation, the patient was severely dyspneic and wheezing with decreased air entry bilaterally. Computed tomography (CT) scan of the chest and neck showed a subglottic lesion that was obstructing the airway (Figure 1). The patient's respiratory status continued to deteriorate, so an elective tracheostomy was performed to secure airway. Later, direct laryngoscopy showed an ulcerated lesion emanating from the left anterior aspect of the subglottis. Biopsy of the ulcerated lesion revealed sheets of malignant melanin containing cells involving the overlying squamous mucosa and extending into the lamina propria. The nuclei were significantly pleomorphic with prominent nucleoli and mitotic figures (Figure 2). The immunoprofile of neoplastic cells was strongly positive for tyrosinase, HMB-45, S-100, and P53 (Figures 3 and 4) and negative for cytokeratin 5/6 and CD34. In order to differentiate the primary from metastatic melanoma, axon 15 BRAF and NRAS testing were performed. The tumor was negative for both of them, which strongly suggested a primary melanoma. Extensive physical exam by a dermatologist and testing including positron emission tomography (PET) scan and CT scans of the body failed to reveal a primary source. The patient was diagnosed with primary malignant melanoma of subglottic mucosa. Total laryngectomy was performed followed by radiation therapy. Five years following the treatment, the patient remains asymptomatic.

## 3. Discussion

Mucosal melanomas represent 1.3% of all melanomas [2, 3]. Majority of patients are white males in their sixth or seventh decade of life with only two reports of Asian individuals [2, 4]. Smoking is a major risk factor [2, 5], but exposure to sunlight, human papilloma virus, chronic irritants, and carcinogenic compounds are also presumed to play a role [2, 6]. Recently, several studies reported that malignant melanomas are related to an altered immune system, and many genes have been speculated to be involved in its pathogenesis but this is not confirmed yet [2].

The patient usually presents with hoarseness of voice, shortness of breath, dysphagia, and sore throat. Differentiation of primary from the secondary lesion may be challenging, especially because of the fact that melanoma may disappear from primary site after metastasis [1, 7, 8]. On gross examination, malignant melanoma may have slate gray, brown, or black pigmentation, which may be a clue to diagnosis. Actual diagnosis cannot be made without histopathological examination of tissue sections [7]. Hematoxylin and eosin staining typically shows pleomorphic, epithelioid, and/or spindle shaped malignant cells extending into adjacent lateral and overlying mucosa. Cells often contain dark brown cytoplasmic and nuclear melanin [8]; however, some lesions are amelanotic and others demonstrate features similar to malignant neoplasm of different origins [7]. Presence of melanoma markers as S-100, HMB-45, Melan-A, and PNL-2 must therefore be demonstrated through immunohistochemical staining to confirm the diagnosis [1, 8]. Electron microscopy may identify the presence of melanosomes or premelanosomes. PET scan and/or magnetic resonance imaging (MRI) can be used to stage primary melanoma [8].

It is obvious that mucosal melanomas are more aggressive and have worse prognosis as compared to their cutaneous counterparts with overall 5-year survival of less than 20% [9]. Poor prognosis is typically associated with early presentation of distant metastases despite adequate locoregional control [10, 11]. Most of mucosal melanomas already have distant micrometastases at the time of diagnosis [1]. The treatment for mucosal melanomas of head and neck, including laryngeal lesions, is complete surgical excision, but sometimes it is difficult because of proximity of tumor to critical structures. Postoperative radiation therapy to the affected area has been shown to improve local control in several retrospective series [10, 12]. Whether this improvement translates to an improvement in prognosis remains unclear. Initial results with traditional chemotherapeutic agents in both cutaneous and mucosal variants of melanoma have been disappointing [12, 13]. Recent studies have shown an involvement of immune system dysregulation in pathophysiology of mucosal melanomas with identification of certain genes like IL17A and CD70 [2]. This can be a major finding towards development of effective adjuvant immunotherapy for the treatment of melanomas.

Primary subglottic melanoma is an exceptionally rare neoplasm with early distant metastases and aggressive fatal course. Early diagnosis and proper treatment are crucial for survival. Physicians should keep a low threshold of suspicion for diagnosis of this rare tumor especially in old age. We believe that every case should be reported for better understanding of this extremely rare disease.
